# A survey of the currently known mast cell mediators with potential relevance for therapy of mast cell-induced symptoms

**DOI:** 10.1007/s00210-023-02545-y

**Published:** 2023-05-27

**Authors:** Gerhard J. Molderings, Lawrence B. Afrin

**Affiliations:** 1 Institute for Human Genetics, University Hospital of Bonn, Venusberg-Campus 1, D-53127 Bonn, Germany; 2 AIM Center for Personalized Medicine, Purchase, New York, USA

**Keywords:** Mast cell, Mast cell mediators, Systemic mast cell activation disease, Mastocytosis

## Abstract

Mast cells (MCs) occupy a central role in immunological as well as non-immunological processes as reflected in the variety of the mediators by which MCs influence other cells. Published lists of MC mediators have all shown only subsets—usually quite small—of the full repertoire. The full repertoire of MC mediators released by exocytosis is comprehensively compiled here for the first time. The compilation of the data is essentially based on the largely cytokine-focused database *COPE*^*®*^, supplemented with data on the expression of substances in human MCs published in several articles, plus extensive research in the PubMed database. Three hundred and ninety substances could be identified as mediators of human MCs which can be secreted into the extracellular space by activation of the MC. This number might still be an underestimate of the actual number of MC mediators since, in principle, all substances produced by MCs can become mediators because of the possibility of their release by diffusion into the extracellular space, mast cell extracellular traps, and intercellular exchange via nanotubules. When human MCs release mediators in inappropriate manners, this may lead to symptoms in any or all organs/tissues. Thus, such MC activation disorders may clinically present with a myriad of potential combinations of symptoms ranging from trivial to disabling or even life-threatening. The present compilation can be consulted by physicians when trying to gain clarity about MC mediators which may be involved in patients with MC disease symptoms refractory to most therapies.

**Supplementary information:**

The online version contains supplementary material available at 10.1007/s00210-023-02545-y.

## Introduction

Mast cells (MCs) are round, about 20 μm diameter cells of the immune system containing cytoplasmic granules variably filled with many messenger substances (mediators). They originate in hematopoietic tissue; white adipose tissue has been identified as a reservoir of MC precursors, too (Poglio et al. 2010). They are resident in all vascularized organs and tissues; the majority are located at the interfaces to the outside world, such as mucous membranes and skin. At these sites, MCs are best positioned to sense when tissues are under attack by potentially harmful pathogens (parasites, bacteria, viruses, venoms) and can act accordingly. In addition, MCs likely have many more underappreciated roles in the human homeostasis of organs that undergo continuous growth and remodeling such as hair follicles and bones, wound healing, disease response, tissue repair, and angiogenesis. They are sensors of hypoxemia, air pressure, vibratory stimuli, and light. In addition, MCs are an integral component of the stress response system (Afrin et al. 2016).

MCs developed more than 500 million years ago (Crivellato et al. 2015), i.e., before the development of adaptive immunity, suggesting that MCs act as effector immune cells and as regulatory immune cells and play central roles in both innate and adaptive immunity (Gri et al. 2012). Through evolution, MCs have become optimized for their already discovered functions such as regulatory control of homeostasis of the organism, potent effector cells of the immune system, and regulation of the functional interaction of the innate and adaptive immune system (Norrby 2022). It seems likely that many other MC mediators with their associated functions remain to be discovered.

The aim of the present survey is to provide all those working in the field of MCs, scientifically and clinically, with a comprehensive compilation of human MC mediators released by exocytosis that can be used as a reference work.

## Methods

The compilation of the data is essentially based on the *COPE*^®^ database (Ibelgaufts 2023), which also contains references that go beyond the references given in the tables herein. This database was last accessed in March 2023. These data were supplemented with data on the expression of proteins in human MCs which had been investigated by, and were published in, Liang et al. (2018), Motakis et al. (2014), Haenisch et al. (2013), Okayama (2005), Halloran et al. (2019), and Babina et al. (2004). In addition, the PubMed database was searched with the phrase human “mast cell*” mediator*. The selective information on the potential effects of the compiled mediators were taken from the *GeneCards*^®^ database (https://www.genecards.org/).

## Results

On the basis of the analyzed databases, 390 substances could be identified (Online Resource 1 and 2) which are formed intracellularly by human MCs and can be secreted by exocytosis into the extracellular space by activation of the MC and can induce effects in effector cells. In studies on murine MCs, another 55 substances have been identified (data not shown) as potential mediators. However, since these substances have not yet been detected in human MCs, they are not further considered as human MC mediators in the following. Each of the 390 potential mediators is able to induce several effects on effector cells (GeneCards^®^). Selected manifestations of MC activation have been linked to specific mediators (Table 1) as an example of using the data from Online Resource 1.

**Table 1:** Selected manifestations of mast cell activation (MCA)

Symptoms	Potential mediators
Dermatologic manifestations	
Urticaria	Histamine
Flush, erythema	Histamine
Angioedema	Histamine, Bradykinin
Hemangiomas, telangiectasias, cherry angiomata, arteriovenous malformations, hemorrhoids, aneurysms, etc.	Probably multiple angiogenic mediators
Wound healing process and keloid formation	Angiopoietin Like 6; Epiregulin
Desquamation in the epidermis	Kallikrein Related Peptidase 5
Respiratory manifestations	
Cough, wheezing	Histamine
Airway inflammation and obstructive dyspnea due to potent smooth muscle contracting activity and proinflammatory activity	Leukotriene C4, D4, E4
Induction of sneezing following exposure to chemical irritants or allergens	Neuromedin B
Anticholinergic symptoms	Acetylcholinesterase
Cardiovascular manifestations	
Hypotension	Adrenomedullin
Hypotension - vasodilator and anti-proliferation agent, counterbalancing the actions of the vasoconstrictor angiotensin II	Angiotensin Converting Enzyme 2
Hypotension - vasodilation and hypotension via bradykinin	Kallikrein 1, Kallikrein Related Peptidase 2, 8, 9, Kininogen 1
Hypotension - plays a key role in mediating cardio-renal homeostasis and vasodilation	Natriuretic Peptide A
Hypotension - vasodilation	Nitric oxide
Hypotension - vasodilation	Platelet activating factor
Hypotension/hypertension - vasodilation at low doses and vasoconstriction at high doses	Prostaglandin D2
Hypertension - a potent vasoconstrictor, affects cardiac contractility and heart rate through its action on the sympathetic nervous system	Angiotensin II, Angiotensinogen
Hypertension - responsible for converting angiotensin I to the vasoactive peptide angiotensin II	chymase 1
Hypertension - potent vasoconstriction	Endothelin 1, 3
Hypertension - vasoconstrictive action	Peptide YY
Hypertension - generation of angiotensin I from angiotensinogen in the plasma, initiating a cascade of reactions which produce hypertension and increased sodium retention by the kidney	Renin
Regulation of heart function	Triiodothyronine, 3-Iodothyroacetic acid; 3-Iodothyroanamine
Atherosclerosis and aortic valve stenosis	Biglycan
Atherosclerosis - disturbed plasma and tissues lipid homeostasis	Apolipoprotein E
Increased erythropoiesis	Erythropoietin, Inhibin subunit α (=activin A)
Gastrointestinal manifestations	
Gastritis – increased gastric acid secretion	Histamine
Anticholinergic symptoms	Acetylcholinesterase
Protective effect - stabilization of the protective mucous gel overlying the gastrointestinal mucosa	Trefoil Factor 1
Enteritis/colitis - important role in the maintenance of intestinal epithelial homeostasis and the promotion of mucosal healing	Milk Fat Globule EGF And Factor V/VIII Domain Containing
Diarrhea – stimulation of colonic smooth muscle contraction	Neuromedin B
Obstipation/dyspepsia - inhibits exocrine pancreatic secretion and inhibitis jejunal and colonic mobility	Peptide YY
Obstipation/dyspepsia/gastroparesis– inhibition of gastrointestinal motility and gastric acid secretion	Trefoil Factor 2
Weight gain or loss - regulator of most hormones of the gastrointestinal tract	Somatostatin
Weight gain or loss - key regulator of energy balance and body weight control	Leptin
Weight gain or loss - disturbed plasma and tissues lipid homeostasis	Apolipoprotein E
Neurologic manifestations	
Increased amyloid precursor protein	A Disintegrin And a Metalloprotease (=ADAM) Domain 9
Increased neuroendocrine stress responses	Adenylate Cyclase Activating Polypeptide 1
Influences on cortical excitability, stress response, food intake, circadian rhythms, and cardiovascular function	Neuropeptide Y
Neurotransmitter and neuromodulator	Neurotensin
Influence on neurogenesis and neuroplasticity associated with learning, memory, depression and chronic pain	VGF Nerve Growth Factor Inducible
Coagulopathic manifestations	
Increased bleeding	
Cleavage of the von Willebrand Factor	ADAM Metallopeptidase With Thrombospondin Type 1 Motif 13
Inhibiting prothrombin activation	Alpha-1-Microglobulin/Bikunin Precursor
May prevent activation of the intrinsic blood coagulation cascade by binding to phospholipids on the surface of damaged cells	Apolipoprotein H
Anticoagulant	Heparin
Inhibition of collagen-induced platelet aggregation	Leukocyte Associated Immunoglobulin Like Receptor 2
Conversion of plasminogen to the fibrinolytic enzyme plasmin	Plasminogen Activator, Tissue Type; Plasminogen Activator, Urokinase
Decreased bleeding/thrombophilia	
Activation of factor XIII	Cathepsin C
Polymerization to form an insoluble fibrin matrix as one of the primary components of blood clots	Fibrinogen α, β, γ-chains
High-molecular-weight kininogenis essential for blood coagulation and assembly of the kallikrein-kinin system	Kininogen 1
Platelet action	Platelet activating factor
Neutralization of heparin on the endothelial surface of blood vessels, thereby inhibiting local antithrombin activity and promoting coagulation	Platelet Factor 4
As well as	
Bind coagulation factor XII leading to its autoactivation	Complement C1q Binding Protein
Inhibition of thrombin, trypsin, plasminogen activator and urokinase	Serpin family A, B, E members
Skeletal manifestations	
Osteolysis	
Increased osteoclast formation	ADAM Metallopeptidase Domain 12
Stimulation of osteoclasts and inhibition of osteoblasts	Activin-A
Autocrine factor which heightens osteoclast formation and bone resorption	Annexin A2
Thiol protease involved in osteoclastic bone resorption	Cathepsin K
Antagonistic effect on osteogenesis due to its direct binding to BMP proteins	DAN (=differential screening-selected gene in neuroblastoma) Domain BMP (=bone morphogenic protein) Antagonist Family Member 5
Negative regulator of bone mineralization	Extracellular Matrix Protein 1
Osteogenesis	
Increased bone growth	Biglycan
Increased osteogenesis	Bone Morphogenetic Protein 2
Ectopic bone formation and promotion of fracture healing	Bone Morphogenetic Protein 7
Regulation of calcium and bone homeostasis	Bone Morphogenetic Protein 8b
Promotes osteogenesis by stimulating the differentiation of mesenchymal progenitors into mature osteoblasts	C-Type Lectin Domain Containing 11A
Stimulates the growth of chondrocytes and osteoblasts	Leukocyte Cell Derived Chemotaxin 2
Pain manifestation	
Inducing pain	
Nociception	Galanin And GMAP Prepropeptide
Headache	Histamine
Direct activation of pain nerve fibers; in the posterior horn of the spinal cord amplification or weakening of pain impulses	Serotonin (5-hydroxytryptamine)
Preprotein of the pain-inducing tachykinin peptide hormone family: substance P, neurokinin A, neuropeptide K, neuropeptide gamma	Tachykinin Precursor 1
Chronic pain	VGF Nerve Growth Factor Inducible
Induction of acute itch	Neuromedin B
Neuromodulation	Nitric oxide
Inhibition of pain	
Preproprotein for the formation of the secreted endogenous opioid peptides beta-neoendorphin, dynorphin, leu-enkephalin, rimorphin, and leumorphin	Prodynorphin
Precursor of β-Endorphin	Proopiomelanocortin
Neurologic manifestations	
Myasthenia	Acetylcholinesterase
Neuroendocrine modulator of pituitary corticotroph function	Cardiotrophin Like Cytokine Factor 1
Mediating the autonomic, behavioral and neuroendocrine responses to stress	Corticotropin Releasing Hormone
Depression of neuronal activity	Cortistatin
Elevated expression of alpha-B crystallin occurs in many neurological diseases	Crystallin Alpha B
Acts as neurotransmitter	Histamine
Modulatory effects on the immune system	
Reduced T-cell activation and proliferation; numbers of hematopoietic stem cells in bone marrow	Activated Leukocyte Cell Adhesion Molecule
Control of the immune response	ADAM Like Decysin 1; Macrophage Migration Inhibitory Factor
Upregulated in multiple inflammatory diseases	Angiopoietin-2
B-cell stimulatory agent	Cardiotrophin Like Cytokine Factor 1
Important role in innate immunity defense against bacteria and viruses	Cathelicidin Antimicrobial Peptide
Activates serine proteases such as elastase, cathepsin G and granzymes A and B	Cathepsin C
Probably involved in the processing of antigenic peptides during MHC class II-mediated antigen presentation; may play a role in activation-induced lymphocyte depletion in the thymus	Cathepsin D
Participates in the killing and digestion of engulfed pathogens; it has bacteriocidal activity	Cathepsin G
Chemotaxis	C-C Motif Chemokine Ligand 1, 2, 3, 4, 4L1, 5,7, 8, 11, 13, 15, 17, 18, 19, 20, 22-25, 28; C17orf99; Ninjurin 1; X-C Motif Chemokine Ligand 1and 2
Controlling the production, differentiation, and function of white cell populations of the blood, the granulocytes and mononuclear phagocytes; promotes the release of pro-inflammatory chemokines	Colony Stimulating Factor 1, 2, 3
Triggering of the complement cascade	Complement C1q A Chain, Complement C1q Binding Protein, Complement C3, C5 Complement Factor D, Complement Factor Properdin
Chemoattractants for various immune cells	C-X3-C Motif Chemokine Ligand 1; C-X-C Motif Chemokine Ligand 1, 2, 3, 5, 8, 9, 10, 11, 12, 14, 16, 17, ISG15 Ubiquitin Like Modifier, Leukocyte Cell Derived Chemotaxin 2, Leukotrien B4
Antibacterial, fungicide and antiviral activities	Defensin Alpha 1, 4, 5 ,6 Beta 1, 4A, 108B, 119; Granulysin; Lysozyme
Inducing cytokine production	High Mobility Group Box 1
Enhances all basic T-cell responses to a foreign antigen	Inducible T Cell Costimulator
Key part of the innate immune response with potent antiviral, antiproliferative and immunomodulatory properties	Interferon Alpha 1, Beta 1, Gamma, Lambda 1-3
Immunoregulation	Interleukin (IL)-1 Alpha, 1 Beta, Interleukin-1 Receptor Antagonist, IL 2-7, 9-11, 12B, 13, 15, 16, 17A, 17C, 17D, 17F, 18, 22, 23 Subunit Alpha, 24, 25, 27, 31, 32, 37
An important component of the non-specific immune system with an antimicrobial activity	Lactotransferrin
Tumor progression/regression by MCA	
Progression	
Important role in tumor progression due to its effect on mRNA production and angiogenesis	Angiogenin
Has been implicated in tumor invasion and metastasis	Cathepsin B, F
Expressed in a significant fraction of human breast cancers, where it could contribute to tumor invasiveness	Cathepsin K
Stimulates the motility of tumor cells and has angiogenic properties, and its expression is upregulated in several kinds of carcinomas	Ectonucleotide Pyrophosphatase/Phosphodiesterase 2
Involved in the growth and proliferation of tumor cells by inducing vasculogenesis	Epidermal Growth Factor-Like Domain Multiple 7
Promotes cancer invasion and metastasis	Kallikrein Related Peptidase 7
Elevated expression of this protein may be associated with cancer cachexia	Inhibin Subunit Beta A
Regression	
Can prevent metastasis by inhibiting vascular growth and tumor cell invasion due to its role as an apoptosis survival factor for vascular endothelial cells	Angiopoietin Like 4
Tumor suppression by stimulation of autophagy and inflammation and an inhibition of angiogenesis and tumorigenesis	Decorin
Inhibits the proliferation of tumor cell	Oncostatin M

**Fig. 1:**
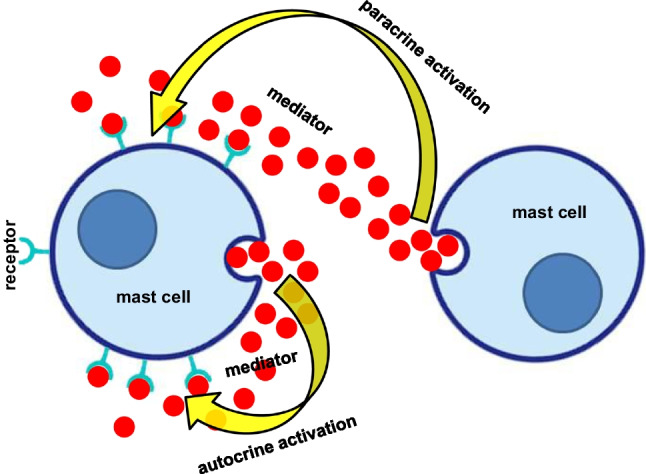
Mast cell activation after mediator (red circles) exocytosis by autocrine and paracrine stimulation of mast cell receptors for this specific released mediator

Understanding the autocrine/paracrine activation of MCs (Fig. 1) is essential for understanding the development of an acute MC mediator release episode (He et al. 2012). Therefore, Table 2 lists all mediators which are likely to induce, via 30 distinct receptor classes, autocrine activation of the releasing MC, and paracrine activation of other MCs in the proximity of the releasing MC. This finding agrees well with the clinical observation of acute to subacute activation phases of MCs beyond anaphylactic reactions. These 30 activating mechanisms are opposed only by seven autocrine/paracrine receptors that can inhibit MC activation (Table 2).

**Table 2:** Facilitatory and inhibitory autocrine regulation of mast cells

Stimulatory receptor	Autocrine ligand
Muscarinic acetylcholine receptor (Haenisch et al. 2013)	Acetylcholine (choline acetyltransferase protein has been found in human skin mast cells; Reinheimer et al. 1998)
Adenosine A2A, A2B, A3 receptors (Haenisch et al. 2013)	Adenosine (Marquardt et al. 1984)
Adrenocorticotropic hormone receptor	Adrenocorticotrophin
Angiotensin-converting enzyme 2	Angiotensin II
C3a, C5a receptors	Complement C3, C5
CXCR1-4 receptors	Chemokines
CD47	Amyloid ß peptides (Niederhoffer et al. 2009)
KIT	Stem cell factor (KIT-ligand)
CD226	Nectin-2 (Bachelet et al. 2006)
CD300	Eosinophilic cationic protein (encoded bei RNASE3)
CRHR-1, 2 receptors	Corticotropin-releasing hormone, urocortin
Cysteinyl receptor 1 and 2 (Jiang et al. 2006)	Leukotrienes
Endothelin receptors types A, B	Endothelin 1, 3
Histamine H_1_- H_2_- H_4_ receptors	Histamine
Interleukin 1 Receptor Type 1 (Jayapal et al. 2006)	Interleukin 1 beta
Interleukin 4 receptor (Haenisch et al. 2013)	Interleukin 4
Interleukin 6 receptor (McHale et al. 2018)	Interleukin 6
Interleukin 10 receptor (Liang et al. 2018)	Interleukin 10
Interleukin 17 receptor (Liang et al. 2018)	Interleukin 17
Interleukin 18 receptor (Haenisch et al. 2013)	Interleukin 18
Low-density lipoprotein-, Very low-density lipoprotein-receptors	Apolipoprotein E
Neurotrophic Receptor Tyrosine Kinase 1 (Peng et al. 2013)	Nerve growth factor
Tachykininreceptor 1, 2 (Le et al. 2016)	Substance P
Neurotensin receptor 1,2 (Alysandratos et al. 2012)	Neurotensin
MRGPRX2 receptor	Opioid peptides
F2R Like Trypsin Receptor 1 (Haenisch et al. 2013)	Proteases
P2Y-, P2X-purinoceptors (Schulman et al. 1999)	ATP
S1P1- S1P2-receptors (Oskeritzian et al. 2008)	S1P
Somatostatin receptor 2 (Haenisch et al. 2013)	Somatostatin
Transforming growth factor beta receptor 1-3 (Haenisch et al. 2013)	Transforming growth factor beta1, beta2
Inhibitory receptors	
Cannabinoid CB2 (CB1) receptor	Anandamide (Bisogno et al. 1997; Braile et al. 2021; )
Glyceraldehyde-3-phosphate dehydrogenase (GAPDH; Haenisch et al. 2013))	Lactoferrin
Interleukin10 receptor (Haenisch et al. 2013)	Il-10
Interleukin 1 Receptor Accessory Protein Like 1	Il-37
Nucleotide converting ectoenzyme E-NPP3 (Tsai and Takeda 2016)	ATP
Peroxisome proliferator-activated receptor gamma (PPAR-γ; Paruchuri et al. 2008)	15-Deoxy-Δ^12,14^-Prostaglandin J_2_ (a metabolite of PGD_2_)
Sialic Acid Binding Ig Like Lectin 9 (Miralda et al. 2023)	Sialic Acid Binding Ig Like Lectin 9 ligand

Two further phenomena could be important for MC activation: first, the possibility of reuptake of mediators released by the MCs for later re-exocytosis. Such reuptake of released mediators may not be accompanied by stimulation of the corresponding receptor because (1) the receptor may still be inactivated due to previous autocrine activation, and (2) reuptake may take place via receptor-independent specific reuptake mechanisms (e.g., transporters). Second, substances originally formed and released by other cells which were taken up and stored by the MCs can potentially act as MC mediators when subsequently released from the MCs (Table 3). The possibility of reuptake or uptake of substances or groups of substances into the MCs which then can act as mediators could be identified for 15 compounds (Table 3).

**Table 3:** Uptake of substances as potential mediators into human mast cells

Lactoferrin (He et al. 2003)	
IL-17A (Noordenbos et al. 2016)	
Extracellular vesicles (Shefler et al. 2021)	
Exosomes (Ekström et al. 2012)	
Fullerene (Dellinger et al. 2010)	
Histamine (Huszti 2003)	
Heparanase (Higashi et al. 2019)	
Thyroid hormones* (via Solute Carrier Family 3 Member 2; Solute Carrier Organic Anion Transporter Family Member 4A1; Solute Carrier Family 16 Member 10; Haenisch et al. 2013)	
Choline, guanidine, histamine, epinephrine, norepinephrine, dopamine* (via Solute Carrier Family 22 Member 1; Haenisch et al. 2013)	
Adenosine* (via Solute Carrier Family 28 Member 3 and Solute Carrier Family 29 Member 1; Haenisch et al. 2013)	
Biogenic amines including serotonin, dopamine, norepinephrine and epinephrine* (via Solute Carrier Family 29 Member 4; Haenisch et al. 2013)	
Gamma-aminobutyric acid* (via Solute Carrier Family 6 Member 13; Solute Carrier Family 36 Member 1; Haenisch et al. 2013)	
Choline* (via Solute Carrier Family 44 Member 1 and 4; Haenisch et al. 2013)	
Prostaglandins D2, E1 and E2, leukotriene C4, thromboxane B2* (via Solute Carrier Organic Anion Transporter Family Member 2B1; Haenisch et al. 2013)	
Prostaglandins E1 and E2, thyroxine and vasopressin* (via Solute Carrier Organic Anion Transporter Family Member 3A1; Haenisch et al. 2013)	

*Deduced from the expression of the respective carrier in HMC1 cells

## Discussion

The central role of MCs in immunological as well as non-immunological processes is reflected by the large number of mediators by which MCs may influence other cells (Lundequist and Pejler 2011). The profile of mediators and cytokines stored or produced de novo in MCs can markedly differ between and even within organs/tissues depending upon a wide array of macro- and micro-environmental factors including antigenic and physical stimuli. Although the number of MC mediators has been assumed to be large, there has not yet been any comprehensive compilation of human MC mediators. In this article, the known human MC mediators are comprehensively compiled for the first time. And indeed, the number of mediators, at least 390, turns out to be extraordinarily high compared to the number of messenger substances known to be formed and released by other cells. However, this number still might substantially underestimate the actual number of MC mediators, once one takes into consideration broader definitions of “mediatorˮ and broader definitions of effector mechanisms than we consider for our present purposes.

MC actions can be targeted very precisely. Occasionally, MCs release pre-stored mediators via classic non-selective whole-MC degranulation (as in anaphylaxis), but this is the exception, not the rule, in MC activation (Theoharides et al. 2007, 2023). Otherwise, anaphylactic reaction would occur consistently in every episode of MC activation, but this is obviously not the case. Rather than wholly degranulate, MCs much more commonly selectively release specific mediators, referred to as differential release (Table 4), i.e., release of the content of individual secretory granules or individual mediators without whole-MC degranulation (Theoharides et al. 1982). This process is distinct from “piecemeal degranulation” that has additionally been reported (Dvorak 2005). MCs can also form synapses for targeted secretion (Table 4).With regard to the possibility that, under certain circumstances, almost all molecules that can be produced by a MC might be able to act as mediators, four release options are of particular interest: (1) diffusion of substances into the extracellular space; (2) release of mRNA, microRNA, and proteins expressed in the MC by secretion of exosomes and vesicles (Savage et al. 2023), some of them containing KIT (Pfeiffer et al. 2022); (3) formation of nanotubules with exchange of intracellular material which seems to be involved in inducing apoptosis in cancer cells (Ahani et al. 2022); and (4) formation of MC extracellular traps (Möllerherm et al. 2016; Table 4). These four mechanisms, by which MCs can use almost any molecule as a mediator, underline the extraordinary role of these cells in our immune system. At the same time, this creates an almost insurmountable hurdle for precisely attributing specific clinical symptoms to specific messenger substances. This problem of assigning (a) certain MC mediator(s) to symptoms is further complicated by the fact that released MC mediators can maintain and enhance MC activation in autocrine and paracrine manners (Fig. 1), and additionally by the possibility of MCs taking up substances from their immediate environment and then re-releasing them. In this context, it has to be noted that MCs are able to survive even complete degranulation followed by regranulation (Iskarpatyoti et al. 2022). Interestingly, MCs have altered granule contents and structure after regranulation, likely depending on the trigger that had induced the degranulation (Friend et al. 1996; Iskarpatyoti et al. 2022, further references therein).

**Table 4:** Forms of communication between mast cells and effector cells

• Mediator release by degranulation	
• Selective exocytotic mediator release	
Untargeted piecemeal degranulation (Theoharides et al. 1982)	
Untargeted by differential release (Theoharides and Douglas 1978; Theoharides et al. 1982; Moon et al. 2014)	
Targeted by synaptic contact with with the target cell (Carroll-Portillo et al. 2012)	
Targeted by mast cell extracellular traps of DNA (Möllerherm et al. 2016; Garcia-Rodriguez et al. 2020)	
• Release of exosomes containing mRNA, microRNA,and proteins (D’Incà and Pucillo 2015; Liang et al. 2018; Kim et al. 2018; Klein and Sagi-Eisenberg 2019; Shefler et al. 2021)	
• Diffusion of mediators into the extracellular space (Kritikou et al. 2016; Chen and Popel 2007)	
• Formation of nanotubules with exchange of intracellular material (Elishmereni et al. 2011; Ahani et al. 2022)	

## Clinical impact

It does not require a great imagination to envision that the very same mechanisms which enable MCs to protect the organism can wreak focused or multisystem havoc when uncontrolled, potentially causing a vast array of diseases, some of which might be quite severe. In this context, primary systemic MC disease (dominantly MC activation syndrome (MCAS)) is of particular interest for at least two reasons: (1) its prevalence of about 20% (Molderings et al. 2013; Maitland et al. 2020) represents a significant socio-economic problem; and (2) due to its epigenetic causation with transgenerational transmission (Molderings 2022), it tends to manifest in successive generations more severely and at steadily earlier ages, creating increasing treatment challenges. Systemic mast cell disease (also presently termed *mast cell activation disease* (MCAD)), in its assorted variants (including systemic mastocytosis and MCAS), is usually driven, at the level of the individual, by multiple stem cell germline and somatic mutations (emerging out of complex interactions between stressor-induced cytokine storms and a genome rendered insufficiently robust, by the aforementioned epigenetic variants, at repairing or eradicating induced mutations) leading directly or indirectly to inappropriate chronic constitutive and reactive activation of the affected MCs (Weinstock et al. 2021). Due to both their widespread distribution and the great heterogeneity of aberrant mediator expression patterns, symptoms may occur in all organs and tissues. Hence, the clinical presentation of MCAD disease is very diverse, with a myriad of combinations of symptoms, ranging in the severity of illness from trivial to disabling and even life-threatening (Afrin et al. 2016).

## Perspective

The present survey of the potential MC mediators in the narrower sense (Online Resource 1 and 2) and broader sense (Table 4), together with the findings of autocrine and paracrine stimulation and the ability of the MC to (re)use substances it takes up as mediators, are not of interest merely to researchers. These tables can be consulted by attending physicians, too, when trying to gain clarity about MC mediators which may be involved in patients with MC disease symptoms which are often resistant to therapy, such as hyper-/hypotension, transient tachyarrhthmias, or migrating pain. Such a procedure might be extraordinarily effective if, based on the available tables and with the help of special computer programs to be developed, all the information contained in relevant databases such as GeneCards^®^, PubMed, *EMBL*’s European Bioinformatics Institute, Embase, Cochrane Library, and others could help link the symptoms in a patient to given mediator expression profiles, thereby hopefully providing personalized therapeutic insights. This might enable the selection of treatments (Molderings et al. 2016) more likely to help patients exhibiting specific MC-mediator-induced symptoms. Ultimately, though, routine performance in the clinical laboratory of MC-specific genome sequencing (using pipelines already in place in many laboratories for sequencing the tumor cells in biopsies, but re-tuned, likely based on strong CD117 expression, to select the MCs in the sample) will be needed to discover not only which mutational profiles reliably correlate with which symptom profiles but also which treatments will best address the phenotypes driven by particular mutational profiles.

### Supplementary information

ESM 1 (XLSX 51 kb)

ESM 2 (DOCX 35 kb)

## Data Availability

Not applicable
