# Supplementary material for: A survey of the currently known mast cell mediators with potential relevance for therapy of mast cell-induced symptoms
Source: Naunyn Schmiedebergs Arch Pharmacol. 2023 May 27;396(11):2881–91. doi: 10.1007/s00210-023-02545-y (PMC10567897; doi:10.1007/s00210-023-02545-y)
Supplement: Supplementary file 2 — (DOCX 35 kb) [file 210_2023_2545_MOESM2_ESM.docx]

**References to Online table 1**

Akoto C, Davies DE, Swindle EJ (2017) Mast cells are permissive for rhinovirus replication: potential implications for asthma exacerbations. Clin Exp Allergy 47:351-360. doi: 10.1111/cea.12879. PMID: 28008678.

Aleman-Muench GR, Soldevila G (2012) When versatility matters: activins/inhibins as key regulators of immunity. Immunol Cell Biol 90:137-148. doi: 10.1038/icb.2011.32. PMID: 21537340.

Amiot L, Vu N, Rauch M, L'Helgoualc'h A, Chalmel F, Gascan H, Turlin B, Guyader D, Samson M (2014) Expression of HLA-G by mast cells is associated with hepatitis C virus-induced liver fibrosis. J Hepatol 60:245-252. doi: 10.1016/j.jhep.2013.09.006. PMID: 24036009.

Artuc M, Steckelings UM, Henz BM (2022) Mast cell-fibroblast interactions: human mast cells as source and inducers of fibroblast and epithelial growth factors. J Invest Dermatol 118:391-395. doi: 10.1046/j.0022-202x.2001.01705.x. PMID: 11874475.

Artuc M, Böhm M, Grützkau A, Smorodchenko A, Zuberbier T, Luger T, Henz BM (2006) Human mast cells in the neurohormonal network: expression of POMC, detection of precursor proteases, and evidence for IgE-dependent secretion of alpha-MSH. J Invest Dermatol 126:1976-981. doi: 10.1038/sj.jid.5700318. PMID: 16675966.

Babina M, Guhl S, Stärke A, Kirchhof L, Zuberbier T, Henz BM (2004) Comparative cytokine profile of human skin mast cells from two compartments--strong resemblance with monocytes at baseline but induction of IL-5 by IL-4 priming. J Leukoc Biol 75:244-252. doi: 10.1189/jlb.0403157. PMID: 14634065.

Bachelet I, Munitz A, Mankutad D, Levi-Schaffer F (2006) Mast cell costimulation by CD226/CD112 (DNAM-1/Nectin-2): a novel interface in the allergic process. J Biol Chem 281:27190-6. doi: 10.1074/jbc.M602359200. PMID: 16831868.

Bianchini R, Koller A, Lang A, Wiesmayr S, Brodowicz B, Mueller E and Kofler B (2013). Expression of the galanin system in immune cells: hypothetical key players of the immune response. Front. Immunol. Conference Abstract: 15th International Congress of Immunology (ICI). doi: 10.3389/conf.fimmu.2013.02.00807

Boyce JA (2005) Eicosanoid mediators of mast cells: receptors, regulation of synthesis, and pathobiologic implications. Chem Immunol Allergy 87:59-79. doi: 10.1159/000087571. PMID: 16107763.

Boyce JA (2007) Mast cells and eicosanoid mediators: a system of reciprocal paracrine and autocrine regulation. Immunol Rev 217:168-185. doi: 10.1111/j.1600-065X.2007.00512.x. PMID: 17498059.

Chen C, Zhang Y, Liu Y, Hang L, Yang J (2022) Expression of Tumor Suppressor SFRP1 Predicts Biological Behaviors and Prognosis: A Potential Target for Oral Squamous Cell Carcinoma. Biomolecules 12:1034. doi: 10.3390/biom12081034. PMID: 35892344.

Cho SH, Yao Z, Wang SW, Alban RF, Barbers RG, French SW, Oh CK (2003) Regulation of activin A expression in mast cells and asthma: its effect on the proliferation of human airway smooth muscle cells. J Immunol 170:4045-452. doi: 10.4049/jimmunol.170.8.4045.

Cho KA, Kim HJ, Kim YH, Park M, Woo SY (2019) Dexamethasone promotes keratinocyte proliferation by triggering keratinocyte growth factor in mast cells. Int Arch Allergy Immunol 179:53-61. doi: 10.1159/000494624. PMID: 30909282.

Chosidow O, Bécherel PA, Piette JC, Arock M, Debré P, Francès C (1998) Tripe palms associated with systemic mastocytosis: the role of transforming growth factor-alpha and efficacy of interferon-alfa. Br J Dermatol 138:698-703. doi: 10.1046/j.1365-2133.1998.02190.x. PMID: 9640384.

Cildir G, Toubia J, Yip KH, Zhou M, Pant H, Hissaria P, Zhang J, Hong W, Robinson N, Grimbaldeston MA, Lopez AF, Tergaonkar V (2019) Genome-wide analyses of chromatin state in human mast cells reveal molecular drivers and mediators of allergic and inflammatory diseases. Immunity 51:949-965.e6. doi: 10.1016/j.immuni.2019.09.021. PMID: 31653482.

Cristinziano L, Poto R, Criscuolo G, Ferrara AL, Galdiero MR, Modestino L, Loffredo S, de Paulis A, Marone G, Spadaro G, Varricchi G (2021) IL-33 and superantigenic activation of human lung mast cells induce the release of angiogenic and lymphangiogenic factors. Cells 10:145. doi: 10.3390/cells10010145. PMID: 33445787.

Dahlen B, Shute J, Howarth P (1999) Immunohistochemical localisation of the matrix metalloproteinases MMP-3 and MMP-9 within the airways in asthma. Thorax 54:590-596. doi: 10.1136/thx.54.7.590. PMID: 10377203.

Domenis R, Pilutti D, Orsaria M, Marzinotto S, Candotti V, Bosisio G, Bulfoni M, Ruaro ME, Di Loreto C, Mea VD, Toffoletti E, Londero AP, Mariuzzi L, Gri G (2018) Expression and modulation of S100A4 protein by human mast cells. Cell Immunol 332:85-93. doi: 10.1016/j.cellimm.2018.08.001. PMID: 30097176.

Dupont A (2008) Properdin in immunity: In vitro and in vivo investigations. Thesis, University of Leicester.

Edwards ST, Cruz AC, Donnelly S, Dazin PF, Schulman ES, Jones KD, Wolters PJ, Hoopes C, Dolganov GM, Fang KC (2005) c-Kit immunophenotyping and metalloproteinase expression profiles of mast cells in interstitial lung diseases. J Pathol 206:279-290. doi: 10.1002/path.1780. PMID: 15887294.

Ehara Y, Yoshida Y, Tahira M, Yamamoto O (2014) The expression of melanoma inhibitory activity on mast cells in child patients with cutaneous mastocytosis. Yonago Acta Med 57:99-101. PMID: 25349464;

Feuser K, Thon KP, Bischoff SC, Lorentz A (2012) Human intestinal mast cells are a potent source of multiple chemokines. Cytokine 58:178-185. doi: 10.1016/j.cyto.2012.01.001. PMID: 22305008.

Foster BM, Langsten KL, Mansour A, Shi L, Kerr BA (2021) Tissue distribution of stem cell factor in adults. Exp Mol Pathol 122:104678. doi: 10.1016/j.yexmp.2021.104678. PMID: 34450114.

Fukuoka Y, Hite MR, Dellinger AL, Schwartz LB (2013) Human skin mast cells express complement factors C3 and C5. J Immunol 191:1827-1834. doi: 10.4049/jimmunol.1202889. PMID: 23833239.

Gilicze A, Kohalmi B, Pocza P, Keszei M, Jaeger J, Gorbe E, Papp Z, Toth S, Falus A, Wiener Z (2007) HtrA1 is a novel mast cell serine protease of mice and men. Mol Immunol 44:2961-8. doi: 10.1016/j.molimm.2007.01.004. PMID: 17292962.

González-Arriagada WA, Santos-Silva AR, Ito FA, Vargas PA, Speight PM, Bingle L, Lopes MA (2012) Expression pattern of PLUNC proteins as an auxiliary tool for the diagnosis of high-grade mucoepidermoid carcinoma of the salivary gland. J Oral Pathol Med 41:589-597. doi: 10.1111/j.1600-0714.2012.01145.x. PMID: 22487508.

Grabbe J, Welker P, Möller A, Dippel E, Ashman LK, Czarnetzki BM (1994) Comparative cytokine release from human monocytes, monocyte-derived immature mast cells, and a human mast cell line (HMC-1). J Invest Dermatol 103:504-508. doi: 10.1111/1523-1747.ep12395649. PMID: 7523530.

Han NR, Ko SG, Moon PD, Park HJ (2021) Chloroquine attenuates thymic stromal lymphopoietin production via suppressing caspase-1 signaling in mast cells. Biomed Pharmacother 141:111835. doi: 10.1016/j.biopha.2021.111835. PMID: 34146852.

Haenisch B, Herms S, Molderings GJ (2013) The transcriptome of the human mast cell leukemia cells HMC-1.2: an approach to identify specific changes in the gene expression profile in KitD816V systemic mastocytosis. Immunol Res 56:155-62. doi: 10.1007/s12026-013-8391-1. PMID: 23504229.

Hara M, Ono K, Wada H, Sasayama S, Matsumori A (2004) Preformed angiotensin II is present in human mast cells. Cardiovasc Drugs Ther 18:415-420. doi: 10.1007/s10557-004-6218-y. PMID: 15770428.

Hasan Q, Rüger BM, Tan ST, Gush J, Davis PF (2000) Clusterin/apoJ expression during the development of hemangioma. Hum Pathol 31:691-697. doi: 10.1053/hupa.2000.7638. PMID: 10872662.

Helske S, Syväranta S, Kupari M, Lappalainen J, Laine M, Lommi J, Turto H, Mäyränpää M, Werkkala K, Kovanen PT, Lindstedt KA (2006) Possible role for mast cell-derived cathepsin G in the adverse remodelling of stenotic aortic valves. Eur Heart J 27:1495-1504. doi: 10.1093/eurheartj/ehi706. PMID: 16401677.

Ho AW, Hatjiharissi E, Ciccarelli BT, Branagan AR, Hunter ZR, Leleu X, Tournilhac O, Xu L, O'Connor K, Manning RJ, Santos DD, Chemaly M, Patterson CJ, Soumerai JD, Munshi NC, McEarchern JA, Law CL, Grewal IS, Treon SP (2008) CD27-CD70 interactions in the pathogenesis of Waldenstrom macroglobulinemia. Blood 112:4683-4689. doi: 10.1182/blood-2007-04-084525. PMID: 18216294.

Hobo A, Harada K, Maeda T, Uchiyama M, Irisawa R, Yamazaki M, Tsuboi R (2020) IL-17-positive mast cell infiltration in the lesional skin of lichen planopilaris: Possible role of mast cells in inducing inflammation and dermal fibrosis in cicatricial alopecia. Exp Dermatol 29:273-277. doi: 10.1111/exd.13816. PMID: 30379356.

Horny HP, Reimann O, Kaiserling E (1988) Immunoreactivity of normal and neoplastic human tissue mast cells. Am J Clin Pathol 89:335-340. doi: 10.1093/ajcp/89.3.335. PMID: 3126643.

Hültner L, Ehrenreich H (2005) Mast cells and endothelin-1: a life-saving biological liaison? Trends Immunol 26:235-8. doi: 10.1016/j.it.2005.03.007. PMID: 15866233.

Huttunen M, Harvima IT (2005) Mast cell tryptase and chymase in chronic leg ulcers: chymase is potentially destructive to epithelium and is controlled by proteinase inhibitors. Br J Dermatol 152:1149-1160. doi: 10.1111/j.1365-2133.2005.06428.x. PMID: 15948975.

Inomata N, Tomita H, Ikezawa Z, Saito H (2005) Differential gene expression profile between cord blood progenitor-derived and adult progenitor-derived human mast cells. Immunol Lett 98:265-271. doi: 10.1016/j.imlet.2004.12.001. PMID: 15860227.

Isogai R, Takahashi M, Aisu K, Horiuti Y, Aragane Y, Kawada A, Tezuka T (2006) The receptor for erythropoietin is present on cutaneous mast cells. Arch Dermatol Res 297:389-394. doi: 10.1007/s00403-005-0615-3. PMID: 16421725.

Ivanova K, I. Stefanov , I. Ivanova , J. Ananiev, M. Gulubova (2021). Ghrelin expression in mast cells of infant lung with respiratory distress syndrome. Acta Medica Bulgarica, XLVIII: 40-45

Jayapal M, Tay HK, Reghunathan R, Zhi L, Chow KK, Rauff M, Melendez AJ (2006) Genome-wide gene expression profiling of human mast cells stimulated by IgE or FcepsilonRI-aggregation reveals a complex network of genes involved in inflammatory responses. BMC Genomics 7:210. doi: 10.1186/1471-2164-7-210. PMID: 16911805.

Jemima EA, Prema A, Thangam EB (2014) Functional characterization of histamine H4 receptor on human mast cells. Mol Immunol 62:19-28. doi: 10.1016/j.molimm.2014.05.007. PMID: 24934979.

Jiang J, Zhan X, Liang T, Chen L, Huang S, Sun X, Jiang W, Chen J, Chen T, Li H, Yao Y, Wu S, Zhu J, Liu C (2022) Dysregulation of SAA1, TUBA8 and monocytes are key factors in ankylosing spondylitis with femoral head necrosis. Front Immunol 12:814278. doi: 10.3389/fimmu.2021.814278. PMID: 35126370.

Jung M, Lord MS, Cheng B, Lyons JG, Alkhouri H, Hughes JM, McCarthy SJ, Iozzo RV, Whitelock JM (2013) Mast cells produce novel shorter forms of perlecan that contain functional endorepellin: a role in angiogenesis and wound healing. J Biol Chem 288:3289-3304. doi: 10.1074/jbc.M112.387811. PMID: 23235151.

Kaieda S, Fujimoto K, Todoroki K, Abe Y, Kusukawa J, Hoshino T, Ida H (2022) Mast cells can produce transforming growth factor β1 and promote tissue fibrosis during the development of Sjögren's syndrome-related sialadenitis. Mod Rheumatol 32:761-769. doi: 10.1093/mr/roab051. PMID: 34915577.

Kakeda M, Schlapbach C, Danelon G, Tang MM, Cecchinato V, Yawalkar N, Uguccioni M (2014) Innate immune cells express IL-17A/F in acute generalized exanthematous pustulosis and generalized pustular psoriasis. Arch Dermatol Res 306:933-938. doi: 10.1007/s00403-014-1488-0. PMID: 25030504.

Kamitani H, Masuzawa H, Sato J, Kanazawa I (1987) Erythropoietin in haemangioblastoma: immunohistochemical and electron microscopy studies. Acta Neurochir (Wien) 85:56-62. doi: 10.1007/BF01402372. PMID: 3300179.

Kamiya S, Ikegami I, Yanagi M, Takaki H, Kamekura R, Sato T, Kobayashi K, Kamiya T, Kamada Y, Abe T, Inoue KI, Hida T, Uhara H, Ichimiya S (2022) Functional Interplay between IL-9 and Peptide YY Contributes to Chronic Skin Inflammation. J Invest Dermatol 142:3222-3231.e5. doi: 10.1016/j.jid.2022.06.021. PMID: 35850207.

Kariyawasam HH, Xanthou G, Barkans J, Aizen M, Kay AB, Robinson DS (2008) Basal expression of bone morphogenetic protein receptor is reduced in mild asthma. Am J Respir Crit Care Med 177:1074-1081. doi: 10.1164/rccm.200709-1376OC. PMID: 18292470.

Kim KW, Kim BM, Won JY, Min HK, Lee KA, Lee SH, Kim HR (2021) Regulation of osteoclastogenesis by mast cell in rheumatoid arthritis. Arthritis Res Ther 23:124. doi: 10.1186/s13075-021-02491-1. PMID: 33882986.

Kinoshita T, Sawai N, Hidaka E, Yamashita T, Koike K (1999) Interleukin-6 directly modulates stem cell factor-dependent development of human mast cells derived from CD34(+) cord blood cells. Blood 94:496-508. PMID: 10397717.

Kirshenbaum AS, Cruse G, Desai A, Bandara G, Leerkes M, Lee CC, Fischer ER, O'Brien KJ, Gochuico BR, Stone K, Gahl WA, Metcalfe DD (2016) Immunophenotypic and ultrastructural analysis of mast cells in Hermansky-Pudlak Syndrome type-1: a possible connection to pulmonary fibrosis. PLoS One 11:e0159177. doi: 10.1371/journal.pone.0159177. PMID: 27459687.

Kobayashi Y, Ueki S, Mahemuti G, Chiba T, Oyamada H, Saito N, Kanda A, Kayaba H, Chihara J (2005) Physiological levels of 15-deoxy-Delta12,14-prostaglandin J2 prime eotaxin-induced chemotaxis on human eosinophils through peroxisome proliferator-activated receptor-gamma ligation. J Immunol 175:5744-5750. doi: 10.4049/jimmunol.175.9.5744. PMID: 16237065.

Koskivirta I, Rahkonen O, Mäyränpää M, Pakkanen S, Husheem M, Sainio A, Hakovirta H, Laine J, Jokinen E, Vuorio E, Kovanen P, Järveläinen H (2006) Tissue inhibitor of metalloproteinases 4 (TIMP4) is involved in inflammatory processes of human cardiovascular pathology. Histochem Cell Biol 126:335-342. doi: 10.1007/s00418-006-0163-8. PMID: 16521002.

Kulka M, Fukuishi N, Rottem M, Mekori YA, Metcalfe DD (2006) Mast cells, which interact with Escherichia coli, up-regulate genes associated with innate immunity and become less responsive to Fc(epsilon)RI-mediated activation. J Leukoc Biol 79:339-350. doi: 10.1189/jlb.1004600. PMID: 16282532.

Kulka M, Sheen CH, Tancowny BP, Grammer LC, Schleimer RP (2008) Neuropeptides activate human mast cell degranulation and chemokine production. Immunology 123:398-410. doi: 10.1111/j.1365-2567.2007.02705.x. PMID: 17922833.

Kulka M, Fukuishi N, Metcalfe DD (2009) Human mast cells synthesize and release angiogenin, a member of the ribonuclease A (RNase A) superfamily. J Leukoc Biol 86:1217-1226. doi: 10.1189/jlb.0908517. PMID: 19625371.

Kvetnoy IM (1999) Extrapineal melatonin: location and role within diffuse neuroendocrine system. Histochem J 31:1-12. doi: 10.1023/a:1003431122334. PMID: 10405817.

Landucci E, Laurino A, Cinci L, Gencarelli M, Raimondi L (2019) Thyroid hormone, thyroid hormone metabolites and mast cells: a less explored issue. Front Cell Neurosci 13:79. doi: 10.3389/fncel.2019.00079. PMID: 30983971.

Lappalainen J, Rintahaka J, Kovanen PT, Matikainen S, Eklund KK (2013) Intracellular RNA recognition pathway activates strong anti-viral response in human mast cells. Clin Exp Immunol 172:121-8. doi: 10.1111/cei.12042. PMID: 23480192.

Larsen LF, Juel-Berg N, Hansen A, Hansen KS, Mills ENC, van Ree R, Rådinger M, Poulsen LK, Jensen BM (2018) No difference in human mast cells derived from peanut allergic versus non-allergic subjects. Immun Inflamm Dis 6:416-427. doi: 10.1002/iid3.226. PMID: 29992767.

Le QT, Gomez G, Zhao W, Hu J, Xia HZ, Fukuoka Y, Katunuma N, Schwartz LB (2011) Processing of human protryptase in mast cells involves cathepsins L, B, and C. J Immunol 187:1912-1918. doi: 10.4049/jimmunol.1001806. PMID: 21742978.

Liang Y, Qiao L, Peng X, Cui Z, Yin Y, Liao H, Jiang M, Li L (2018) The chemokine receptor CCR1 is identified in mast cell-derived exosomes. Am J Transl Res 10:352-367. PMID: 29511430.

Liu H, Tan J, Liu J, Feng H, Pan D (2020) Altered mast cell activity in response to rhinovirus infection provides novel insight into asthma. J Asthma 57:459-467. doi: 10.1080/02770903.2019.1585870. PMID: 30882256.

Liu W, Xu L, Liang X, Liu X, Zhao Y, Ma C, Gao L (2020) Tim-4 in health and disease: friend or foe? Front Immunol 11:537. doi: 10.3389/fimmu.2020.00537. PMID: 32300343.

Lundequist A, Pejler G (2011) Biological implications of preformed mast cell mediators. Cell Mol Life Sci 68:965-975. doi: 10.1007/s00018-010-0587-0. PMID: 21069421.

Luscan A, Shackleford G, Masliah-Planchon J, Laurendeau I, Ortonne N, Varin J, Lallemand F, Leroy K, Dumaine V, Hivelin M, Borderie D, De Raedt T, Valeyrie-Allanore L, Larousserie F, Terris B, Lantieri L, Vidaud M, Vidaud D, Wolkenstein P, Parfait B, Bièche I, Massaad C, Pasmant E (2014) The activation of the WNT signaling pathway is a hallmark in neurofibromatosis type 1 tumorigenesis. Clin Cancer Res 20:358-371. doi: 10.1158/1078-0432.CCR-13-0780. PMID: 24218515.

Maaninka K, Lappalainen J, Kovanen PT (2013) Human mast cells arise from a common circulating progenitor. J Allergy Clin Immunol 132:463-469.e3. doi: 10.1016/j.jaci.2013.02.011. PMID: 23582567.

Marone G, Varricchi G, Loffredo S, Granata F (2016) Mast cells and basophils in inflammatory and tumor angiogenesis and lymphangiogenesis. Eur J Pharmacol 778:146-51. doi: 10.1016/j.ejphar.2015.03.088. PMID: 25941082.

Marquardt DL, Gruber HE, Wasserman SI (1984) Adenosine release from stimulated mast cells. Proc Natl Acad Sci U S A 81:6192-6196. doi: 10.1073/pnas.81.19.6192. PMID: 6435127.

Maseruka H, Ridgway A, Tullo A, Bonshek R (2000) Developmental changes in patterns of expression of tenascin-C variants in the human cornea. Invest Ophthalmol Vis Sci 41:4101-4107. PMID: 11095602.

Mashiko S, Bouguermouh S, Rubio M, Baba N, Bissonnette R, Sarfati M (2015) Human mast cells are major IL-22 producers in patients with psoriasis and atopic dermatitis. J Allergy Clin Immunol 136:351-359.e1. doi: 10.1016/j.jaci.2015.01.033. PMID: 25792465.

Mathivanan S, Lim JW, Tauro BJ, Ji H, Moritz RL, Simpson RJ (2010) Proteomics analysis of A33 immunoaffinity-purified exosomes released from the human colon tumor cell line LIM1215 reveals a tissue-specific protein signature. Mol Cell Proteomics 9:197-208. doi: 10.1074/mcp.M900152-MCP200. PMID: 19837982;.

McCurdy JD, Olynych TJ, Maher LH, Marshall JS (2003) Cutting edge: distinct Toll-like receptor 2 activators selectively induce different classes of mediator production from human mast cells. J Immunol 170:1625-1629. doi: 10.4049/jimmunol.170.4.1625. PMID: 12574323.

McHale C, Mohammed Z, Gomez G (2019) Human skin-derived mast cells spontaneously secrete several angiogenesis-related factors. Front Immunol 10:1445. doi: 10.3389/fimmu.2019.01445. PMID: 31293594.

Mitamura Y, Nunomura S, Furue M, Izuhara K (2020) IL-24: A new player in the pathogenesis of pro-inflammatory and allergic skin diseases. Allergol Int 69:405-411. doi: 10.1016/j.alit.2019.12.003. PMID: 31980374.

Molderings GJ (2010) Mast cell function in physiology and pathophysiology. Biotrend Rev 5:1-9

Molnár V, Érsek B, Wiener Z, Tömböl Z, Szabó PM, Igaz P, Falus A (2012) MicroRNA-132 targets HB-EGF upon IgE-mediated activation in murine and human mast cells. Cell Mol Life Sci 69:793-808. doi: 10.1007/s00018-011-0786-3. PMID: 21853268.

Mori K, Kitayama J, Aoki J, Kishi Y, Shida D, Yamashita H, Arai H, Nagawa H (2007) Submucosal connective tissue-type mast cells contribute to the production of lysophosphatidic acid (LPA) in the gastrointestinal tract through the secretion of autotaxin (ATX)/lysophospholipase D (lysoPLD). Virchows Arch 451:47-56. doi: 10.1007/s00428-007-0425-4. PMID: 17554559.

Morita H, Arae K, Unno H, Miyauchi K, Toyama S, Nambu A, Oboki K, Ohno T, Motomura K, Matsuda A, Yamaguchi S, Narushima S, Kajiwara N, Iikura M, Suto H, McKenzie AN, Takahashi T, Karasuyama H, Okumura K, Azuma M, Moro K, Akdis CA, Galli SJ, Koyasu S, Kubo M, Sudo K, Saito H, Matsumoto K, Nakae S (2015) An interleukin-33-mast cell-interleukin-2 axis suppresses papain-induced allergic inflammation by promoting regulatory T cell numbers. Immunity 43:175-186. doi: 10.1016/j.immuni.2015.06.021. PMID: 26200013

Motakis E, Guhl S, Ishizu Y, Itoh M, Kawaji H, de Hoon M, Lassmann T, Carninci P, Hayashizaki Y, Zuberbier T, Forrest AR, Babina M; FANTOM consortium (2014) Redefinition of the human mast cell transcriptome by deep-CAGE sequencing. Blood 123:e58-67. doi: 10.1182/blood-2013-02-483792. PMID: 24671954.

Ng MF (2010) The role of mast cells in wound healing. *Int Wound J* 7:55-61. doi: 10.1111/j.1742-481X.2009.00651.x. PMID: 20409251.

Niyonsaba F, Ushio H, Hara M, Yokoi H, Tominaga M, Takamori K, Kajiwara N, Saito H, Nagaoka I, Ogawa H, Okumura K (2010) Antimicrobial peptides human beta-defensins and cathelicidin LL-37 induce the secretion of a pruritogenic cytokine IL-31 by human mast cells. J Immunol 184:3526-3534. doi: 10.4049/jimmunol.0900712. PMID: 20190140.

Noordenbos T, Blijdorp I, Chen S, Stap J, Mul E, Cañete JD, Lubberts E, Yeremenko N, Baeten D (2016) Human mast cells capture, store, and release bioactive, exogenous IL-17A. J Leukoc Biol 100:453-462. doi: 10.1189/jlb.3HI1215-542R. PMID: 27034403.

Nouri-Aria KT, Pilette C, Jacobson MR, Watanabe H, Durham SR (2005) IL-9 and c-Kit+ mast cells in allergic rhinitis during seasonal allergen exposure: effect of immunotherapy. J Allergy Clin Immunol 116:73-79. doi: 10.1016/j.jaci.2005.03.011. PMID: 15990777.

Okayama Y (2005) Mast cell-derived cytokine expression induced via Fc receptors and Toll-like receptors. Chem Immunol Allergy 87:101-110. doi: 10.1159/000087574. PMID: 16107766.

Okragly AJ, Morin SM, DeRosa D, Martin AP, Johnson KW, Johnson MP, Benschop RJ (2018) Human mast cells release the migraine-inducing factor pituitary adenylate cyclase-activating polypeptide (PACAP). Cephalalgia 38:1564-1574. doi: 10.1177/0333102417740563. PMID: 29103295.

Peng WM, Maintz L, Allam JP, Raap U, Gütgemann I, Kirfel J, Wardelmann E, Perner S, Zhao W, Fimmers R, Walgenbach K, Oldenburg J, Schwartz LB, Novak N (2013) Increased circulating levels of neurotrophins and elevated expression of their high-affinity receptors on skin and gut mast cells in mastocytosis. Blood 122:1779-1788. doi: 10.1182/blood-2012-12-469882. PMID: 23869086.

Pilkington SM, Barron MJ, Watson REB, Griffiths CEM, Bulfone-Paus S (2019) Aged human skin accumulates mast cells with altered functionality that localize to macrophages and vasoactive intestinal peptide-positive nerve fibres. Br J Dermatol 180:849-858. doi: 10.1111/bjd.17268. PMID: 30291626.

Prasad P, Yanagihara AA, Small-Howard AL, Turner H, Stokes AJ (2008) Secretogranin III directs secretory vesicle biogenesis in mast cells in a manner dependent upon interaction with chromogranin A. J Immunol 181:5024-5034. doi: 10.4049/jimmunol.181.7.5024. PMID: 18802106.

Prevete N, Staiano RI, Granata F, Detoraki A, Necchi V, Ricci V, Triggiani M, De Paulis A, Marone G, Genovese A (2013) Expression and function of angiopoietins and their tie receptors in human basophils and mast cells. J Biol Regul Homeost Agents 27:827-839. PMID: 24152847.

Rahkola D, Laitala J, Siiskonen H, Pelkonen J, Harvima IT (2019) Mast cells are a marked source for complement C3 products that associate with increased CD11b-positive cells in keratinocyte skin carcinomas. Cancer Invest 37:73-84. doi: 10.1080/07357907.2019.1565765. PMID: 30689427.

Reinheimer T, Vogel P, Racké K, Bittinger F, Kirkpatrick CJ, Saloga J, Knop J, Wessler I (1998) Non-neuronal acetylcholine is increased in chronic inflammation like atopic dermatitis. *Naunyn-Schmiedeberg’s Arch Pharmacol* (Suppl) 358: R87

Richter B, Roslind A, Hesse U, Nordling J, Johansen JS, Horn T, Hansen AB (2010) YKL-40 and mast cells are associated with detrusor fibrosis in patients diagnosed with bladder pain syndrome/interstitial cystitis according to the 2008 criteria of the European Society for the Study of Interstitial Cystitis. Histopathology 57:371-383. doi: 10.1111/j.1365-2559.2010.03640.x. PMID: 20840668.

Rönnberg E, Calounova G, Sutton VR, Trapani JA, Rollman O, Hagforsen E, Pejler G (2014) Granzyme H is a novel protease expressed by human mast cells. Int Arch Allergy Immunol 165:68-74. doi: 10.1159/000368403. PMID: 25342632.

Rüger B, Dunbar PR, Hasan Q, Sawada H, Kittelberger R, Greenhill N, Neale TJ (1994) Human mast cells produce type VIII collagen in vivo. Int J Exp Pathol 75:397-404. PMID: 7734329.

Samitas K, Zervas E, Vittorakis S, Semitekolou M, Alissafi T, Bossios A, Gogos H, Economidou E, Lötvall J, Xanthou G, Panoutsakopoulou V, Gaga M (2011) Osteopontin expression and relation to disease severity in human asthma. Eur Respir J 37:331-341. doi: 10.1183/09031936.00017810. PMID: 20562127.

Sasai Y, Ishibashi M (1975) Histochemical demonstration of hyaluronic acid in human dermal mast cells. Tohoku J Exp Med 116:285-293. doi: 10.1620/tjem.116.285. PMID: 52204.

Sayama K, Diehn M, Matsuda K, Lunderius C, Tsai M, Tam SY, Botstein D, Brown PO, Galli SJ (2002) Transcriptional response of human mast cells stimulated via the Fc(epsilon)RI and identification of mast cells as a source of IL-11. BMC Immunol 3:5. doi: 10.1186/1471-2172-3-5. PMID: 12079505.

Schwartz LB, Lewis RA, Seldin D, Austen KF (1981) Acid hydrolases and tryptase from secretory granules of dispersed human lung mast cells. J Immunol 126:1290-1294. PMID: 7009736.

Shakoory B, Fitzgerald SM, Lee SA, Chi DS, Krishnaswamy G (2004) The role of human mast cell-derived cytokines in eosinophil biology. J Interferon Cytokine Res 24:271-281. doi: 10.1089/107999004323065057. PMID: 15153310.

Siddhuraj P, Clausson CM, Sanden C, Alyamani M, Kadivar M, Marsal J, Wallengren J, Bjermer L, Erjefält JS (2021) Lung mast cells have a high constitutive expression of carboxypeptidase A3 mRNA that is independent from granule-stored CPA3. Cells 10:309. doi: 10.3390/cells10020309. PMID: 33546258.

Sime W (2007) The diverse role of laminin isoforms in neuronal cells, human mast cells and blood platelets. Karolinska University Press, Stockholm, Sweden. Thesis. ISBN 91-7357-122-7.

Solinski HJ, Kriegbaum MC, Tseng PY, Earnest TW, Gu X, Barik A, Chesler AT, Hoon MA (2019) Nppb neurons are sensors of mast cell-induced itch. Cell Rep 26:3561-3573.e4. doi: 10.1016/j.celrep.2019.02.089. PMID: 30917312.

Syenina A, Saron WAA, Jagaraj CJ, Bibi S, Arock M, Gubler DJ, Rathore APS, Abraham SN, St John AL (2020) Th1-polarized, Dengue virus-activated human mast cells induce endothelial transcriptional activation and permeability. Viruses 12:1379. doi: 10.3390/v12121379. PMID: 33276578.

Tada H, Nishioka T, Takase A, Numazaki K, Bando K, Matsushita K (2019) Porphyromonas gingivalis induces the production of interleukin-31 by human mast cells, resulting in dysfunction of the gingival epithelial barrier. Cell Microbiol 21:e12972. doi: 10.1111/cmi.12972. Epub 2018 Nov 28. PMID: 30423602.

Taildeman J, Pérez-Novo CA, Rottiers I, Ferdinande L, Waeytens A, De Colvenaer V, Bachert C, Demetter P, Waelput W, Braet K, Cuvelier CA (2009) Human mast cells express leptin and leptin receptors. Histochem Cell Biol 131:703-711. doi: 10.1007/s00418-009-0575-3. PMID: 19241089.

Tebroke J, Lieverse JE, Säfholm J, Schulte G, Nilsson G, Rönnberg E (2019) Wnt-3a induces cytokine release in human mast cells. Cells 8:1372. doi: 10.3390/cells8111372. PMID: 31683769.

Theoharides TC, Donelan JM, Papadopoulou N, Cao J, Kempuraj D, Conti P (2004) Mast cells as targets of corticotropin-releasing factor and related peptides. Trends Pharmacol Sci 25:563-568. doi: 10.1016/j.tips.2004.09.007. PMID: 15491778.

Toyoda M, Nakamura M, Nakada K, Nakagawa H, Morohashi M (2005) Characteristic alterations of cutaneous neurogenic factors in photoaged skin. Br J Dermatol 153 Suppl 2:13-22. doi: 10.1111/j.1365-2133.2005.06965.x. PMID: 16280017.

Trentin Brum S, Demasi AP, Fantelli Stelini R, Cintra ML, Cavalcanti de Araujo V, Borges Soares A (2019) Endoglin is highly expressed in human mast cells. Appl Immunohistochem Mol Morphol 27:613-617. doi: 10.1097/PAI.0000000000000668. PMID: 29734249.

Tsutsui-Takeuchi M, Ushio H, Fukuda M, Yamada T, Niyonsaba F, Okumura K, Ogawa H, Ikeda S (2015) Roles of retinoic acid-inducible gene-I-like receptors (RLRs), Toll-like receptor (TLR) 3 and 2'-5' oligoadenylate synthetase as viral recognition receptors on human mast cells in response to viral infection. Immunol Res 61:240-249. doi: 10.1007/s12026-014-8617-x. PMID: 25550087;.

van Schaarenburg RA, Suurmond J, Habets KL, Brouwer MC, Wouters D, Kurreeman FA, Huizinga TW, Toes RE, Trouw LA (2016) The production and secretion of complement component C1q by human mast cells. Mol Immunol 78:164-170. doi: 10.1016/j.molimm.2016.09.001. PMID: 27648858.

Vysniauskaite M, Hertfelder HJ, Oldenburg J, Dreßen P, Brettner S, Homann J, Molderings GJ (2015) Determination of plasma heparin level improves identification of systemic mast cell activation disease. PLoS One 10:e0124912. doi: 10.1371/journal.pone.0124912. PMID: 25909362.

Wakahara S, Fujii Y, Nakao T, Tsuritani K, Hara T, Saito H, Ra C (2001) Gene expression profiles for Fc epsilon RI, cytokines and chemokines upon Fc epsilon RI activation in human cultured mast cells derived from peripheral blood. Cytokine 16:143-152. doi: 10.1006/cyto.2001.0958. PMID: 11792124.

Wang G, Fan WT, Zhang Z, Huang SG (2018) Expression of matrix metalloproteinase-8 and matrix metalloproteinase-13 in mast cells of human periapical lesions. Int J Clin Exp Pathol 11:2530-2536. PMID: 31938366.

Wang YT, Wang H, Wang FL, Qian XM, Zhuang SF, Yang MX, Liu CX (2018) Effect of IFN-λ2 on combined allergic rhinitis with nasal polyps. Eur Rev Med Pharmacol Sci 22:1588-1594. doi: 10.26355/eurrev_201803_14563. PMID: 29630100.

Wang Z, Franke K, Zuberbier T, Babina M (2022) Cytokine stimulation by MRGPRX2 occurs with lower potency than by FcεRI aggregation but with similar dependence on the extracellular signal-regulated kinase 1/2 module in human skin mast cells. J Invest Dermatol 142:414-424.e8. doi: 10.1016/j.jid.2021.07.153. PMID: 34329659.

Watts MM, Marie Ditto A (2019) Anaphylaxis. Allergy Asthma Proc 40:453-456. doi: 10.2500/aap.2019.40.4270. PMID: 31690393.

Westin U, Polling A, Ljungkrantz I, Ohlsson K (1999) Identification of SLPI (secretory leukocyte protease inhibitor) in human mast cells using immunohistochemistry and in situ hybridisation. Biol Chem 380:489-493. doi: 10.1515/BC.1999.063. PMID: 10355635.

Wezel A, Lagraauw HM, van der Velden D, de Jager SC, Quax PH, Kuiper J, Bot I (2015) Mast cells mediate neutrophil recruitment during atherosclerotic plaque progression. Atherosclerosis 241:289-296. doi: 10.1016/j.atherosclerosis.2015.05.028. PMID: 26062988.

Wu ML, Liu FL, Sun J, Li X, He XY, Zheng HY, Zhou YH, Yan Q, Chen L, Yu GY, Chang J, Jin X, Zhao J, Chen XW, Zheng YT, Wang JH (2021) SARS-CoV-2-triggered mast cell rapid degranulation induces alveolar epithelial inflammation and lung injury. Signal Transduct Target Ther 6:428. doi: 10.1038/s41392-021-00849-0. PMID: 34921131.

Yao W, Yang H, Yin N, Ding G (2014) Mast cell-nerve cell interaction at acupoint: modeling mechanotransduction pathway induced by acupuncture. Int J Biol Sci 10:511-519. doi: 10.7150/ijbs.8631. PMID: 24910530.

Yeo D, Hwang SJ, Song YS, Lee HJ (2021) Humulene inhibits acute gastric mucosal injury by enhancing mucosal integrity. Antioxidants (Basel) 10:761. doi: 10.3390/antiox10050761. PMID: 34064830.

Yu Y, Blokhuis BRJ, Diks MAP, Keshavarzian A, Garssen J, Redegeld FA (2018) Functional inhibitory Siglec-6 is upregulated in human colorectal cancer-associated mast cells. Front Immunol 9:2138. doi: 10.3389/fimmu.2018.02138. PMID: 30294327.

Zebrowska A, Wagrowska-Danilewicz M, Danilewicz M, Stasikowska-Kanicka O, Kulczycka-Siennicka L, Wozniacka A, Waszczykowska E (2014) Mediators of mast cells in bullous pemphigoid and dermatitis herpetiformis. Mediators Inflamm 2014:936545. doi: 10.1155/2014/936545. PMID: 25400334.

Żelechowska P, Pastwińska J, Brzezińska-Błaszczyk E, Agier J (2021) Do mast cells contribute to the antifungal host defense? Cells 10:2510. doi: 10.3390/cells10102510. PMID: 34685489.

Zhan M, Zheng W, Jiang Q, Zhao Z, Wang Z, Wang J, Zhang H, He S (2017) Upregulated expression of substance P (SP) and NK1R in eczema and SP-induced mast cell accumulation. Cell Biol Toxicol 33:389-405. doi: 10.1007/s10565-016-9379-0. PMID: 28154998.
